# The Future of Voluntary Hospitals

**Published:** 1923-07

**Authors:** H. J. Waring

**Affiliations:** Vice-Chancellor of the University of London


### THE FUTURE OF VOLUNTARY HOSPITALS'

By Mr. H. J. WARING, F.R.C.S., Vice-Chancellor of the University of London.

THE term "Voluntary Hospital" in the public mind has varying significations. By the medical man and by many of the public, a "Voluntary Hospital" is understood to be a public medical institution supported and maintained by charitable benefaction or voluntary subscriptions and donations, to which the suffering and necessitous poor can be admitted at all times for treatment. There are three varieties of Voluntary Hospitals: General Hospitals, Special Hospitals and Cottage Hospitals, so-called. In the past, the medical staff of a Voluntary Hospital has given its services either entirely gratuitously or, in a few instances, each member of the staff has received a small honorarium for out-of-pocket expenses. It has always been understood, however, by the honorary medical staff of a Voluntary Hospital that the services given in this honorary capacity were to be for the necessitous poor, and not for patients for whom or from whom the hospital has received monetary consideration for treatment. Some hospitals in this way receive considerable sums of money. In my own hospital—St. Bartholomew's—last year the amount of money received in this way was nearly £30,000, about one-fifth of the annual hospital expenditure. On this account the position of the medical staff and finance ought to be reconsidered.

### The "Hospital Classes."

Of recent years since the war the financial condition of a large number of hospital patients has been much improved, so much so that a considerable number are able to make monetary contributions to the hospital authorities either towards or for their hospital accommodation, maintenance and nursing. At the same time an increasing number of the middle and professional classes, who years ago were well able to afford medical treatment either in their own homes or in private nursing homes and hospitals, have been compelled on account of their diminished pecuniary resources to apply to the Voluntary Hospitals for treatment. Many members of this class are able to pay either the whole or part of the cost of their hospital accommodation, maintenance and nursing, but usually nothing for their treatment. A number of these, however, can pay something for their treatment, and it has been my experience of recent years that a gradually increasing number of patients of the middle and professional classes and the so-called "new poor" are extremely desirous of obtaining hospital treatment at reasonable rates. Also many of the wealthier classes desire to be allowed to enter Voluntary General Hospitals for treatment, but with better accommodation than that provided for those in the Charity wards. Owing, however, to the origin, the constitution and the arrangement of the accommodation in many of our hospitals this is not generally possible. In some cases this is due to the fact that the hospital buildings have been primarily provided for the necessitous poor, and not for patients who are able to pay for their treatment.

### Accommodation for All Classes.

The practical result as regards the general institution of payment by patients for or towards hospital maintenance and treatment is that in order to be fair to all classes of the community every Voluntary Hospital should provide suitable accommodation for all classes of the community who wish to avail themselves of its resources. In order to do this it will be necessary for most Voluntary Hospitals to build new accommodation suitable to this purpose, since in nearly all cases the present accommodation is designed for the necessitous poor. I have had prepared a series of plans of a suitable addition to a Voluntary General Hospital for such a purpose. These plans are designed for a hospital paying block able to accommodate 100 patients. In order to adapt these plans to any individual voluntary hospital it would be necessary in a large number of instances to build either smaller or larger buildings according to the size of the hospital and the requirements of the community where the hospital is situated.

### Advantages of Paying Patients.

The advantages which would be gained by the provision of accommodation for paying patients in a Voluntary Hospital would be as follows:—To Patients.—Members of the upper, middle and professional classes would be able readily to obtain institutional investigation and treatment of the same high standard as that which they provide or assist in providing for the necessitous poor. To Hospitals.—The special departments, both for investigation and treatment, of many hospitals could be more fully and continuously utilised than at the present time, especially in hospitals where such departments are only open on two or three days or half-days per week. Departments of pathology, X-ray, electro-therapeutics and mechano-therapeutics are examples of this nature. The overhead charges for general administration would not of necessity be increased, or in any case would not be proportionately increased; a portion of such charges could legitimately be transferred to the paying department, which could be made a source of profit to the hospital. To Hospital Staff.—Members of hospital staffs would be able to have some of their own patients in the private rooms or paying department, and thus save themselves considerable time in going from one private hospital or nursing home to another. To Staff and Patients.—Resident medical officers could be appointed to a paying department of this kind, whose services would be available for patients in the absence of the patient's own medical man. The advantages provided by the establishment of a paying department of the kind mentioned would, I think, be most appreciated in connection with maternity and surgery.

### The Panel System.

The Government panel system appears to me to be very insufficient. It provides medical assistance for a certain proportion of the community, in the main for slight ailments. When panel patients suffer from affections of a serious nature, it is nearly always necessary for such patients to be sent to one or other of the Voluntary Hospitals, where they are accommodated, maintained and treated by the hospital and its staff, usually without any payment from Government sources. The deficiencies of the panel system appear to be primarily due to the fact that in designing such a system it was assumed that most patients can be cured of their diseases by the administration of drugs in one form or another. In order to illustrate the amount of work which is done by a Voluntary Hospital, for "Insured Persons," at St. Bartholomew's during the past year—1922—I find that 71·8 per cent. adult males and 31·2 per cent. adult females of the total adult persons admitted to the wards were of the "Insured Class"; that is to say, 51 per cent. of the total adults who received in-patient treatment. If this amount of work done by one Voluntary Hospital for "Insured Persons" is a fair example of what is done by other Voluntary Hospitals, and I have no reason to doubt that this is so, surely the State ought to give them material help in return for the benefits given to a class for whom it claims to provide medical assistance.

### Provision of Clinical Laboratories.

It is essential that every hospital, or in the case of large hospitals individual units, should have in direct proximity to patients' rooms or wards a well-equipped clinical laboratory where ordinary clinical pathological investigations can be carried out. The provision of a clinical pathological laboratory in connection with a small hospital and the appointment of a pathologist has not generally been adopted, owing to the fact that there is not sufficient work to employ fully a pathological member of the staff. If, however, patients of all classes can have recourse to hospitals for the investigation and treatment of their diseases, pathologists and other special investigators would have quite sufficient work to employ their time, and sufficient funds would be available for their payment.

### Payment of Medical Staffs.

In the past, resident members of the hospital staff and also junior members of visiting staff have not been paid adequately for their services. In the case of the resident staff the view is often held that it is a teaching post, and consequently that, as the occupant is completing his education, payment for his services is not necessary. It must be remembered, however, that a newly qualified medical man has already spent in all probability five or six years in getting his qualification, and that, having obtained it, it seems only right and proper that he should be properly paid for the services he renders to the hospital. The same statement also holds as regards the junior members of the visiting staff.

---

### Hospital's Record Income.

The Earl of Chesterfield, presiding at the annual meeting of St. John's Hospital for Diseases of the Skin, Leicester Square, stated that the provision of a nurses' home for the in-patients' department at Uxbridge Road was now an accomplished fact. While the total income reached a record figure in the history of the hospital, there had again been a fall in the number and amount of the annual subscriptions.

### AFTER-CARE OF HOSPITAL PATIENTS.*

By MISS A. E. CUMMINS, Lady Almoner of St. Thomas's Hospital.

LET me give you just four examples of cases which come before us: The working men who must down tools for a time with a large family on his hands; the single woman who is pregnant with nowhere to go; the woman with gastric trouble who will need special diet; the girl who has venereal disease and is sharing a small bedroom with three younger sisters. To give them hospital treatment will be a farce, no matter how scientific, unless there are radical changes in their homes. Inside the hospital there are the doctors, devoting long hours to considering, diagnosing and treating their patients. Is it too much to say that much of their skill will be wasted, and that they are justified in asking for the services of an intelligent social worker to co-operate with them, to bring their trained knowledge of economics and social science to bear on each of those lives?

### An Abnormal Setting.

That surgical instruments, convalescence and diet are often the necessary adjuncts to hospital treatment all will agree; but to-night we are going beyond this, and considering whether for a large number of patients home care and consideration is not necessary if the full value from the modern methods and the scientific treatment of our hospitals is to be obtained. In private practice, the doctor visits his patient in his or her normal surroundings, and thus can consider all aspects before treating—economics, environment and way of living, &c. In the hospital the patient has an abnormal setting. In a neurological department it is no infrequent occurrence for the physician to delay diagnosis and treatment until after a report on social conditions is obtained. In a venereal department the women and girls need much help and support, if only to enable them to continue the regular attendance on which so much depends, and beyond that there is the vast field for helping those who feel they have taken a wrong turning and would be willing to start again on better and healthier lines if they had the means to do so.

### A Part of Hospital Treatment.

Now I want to submit a further consideration—that this social service should have its headquarters at the hospital, should, in fact, be a part of hospital treatment, a partner in the team work consisting of doctor, nurse, social worker and patient. Surely some centre must exist inside the hospital to act as the link between the medical world and the philanthropic agencies, and no special work for hospital patients is possible without the closest co-operation with all the forces which exist for the furtherance of public health or the alleviation of distress. Secondly, I would boldly say to those who consider that such work should not be provided from hospital funds, in that it cannot be termed medical work, that it is just as much a part of modern hospital equipment as the dispensary, porter's lodge or registrar's office.

---

* A brief summary of a paper read at the Sheffield Hospital Conference.

